# The complete plastid genome of *Phoenix canariensis* Chabaud (Arecaceae) and phylogenetic analysis

**DOI:** 10.1080/23802359.2020.1852900

**Published:** 2021-01-13

**Authors:** Bowen Zou, Wanwan Long, Li He Yang Wu

**Affiliations:** College of Life Science, Jinggangshan University, Ji’an, Jiangxi, China

**Keywords:** Arecaceae, *P. canariensis*, plastid genome, phylogenetic

## Abstract

*Phoenix canariensis* Chabaud is a vital ornamental and widely planted in the urban landscape of China. In this study, we reported the complete chloroplast genome (cpDNA) of *P. canariensis*, which is 158,477 bp in length, including a large single copy region (LSC) of 86,189 bp, a small single copy region (SSC) of 17,704 bp, and a pair of inverted repeats (IR) regions of 27,292 bp inserted between LSC and SSC. 132 genes are encoded, including 86 protein-encoding genes, 8 ribosomal RNA genes, and 38 transfer RNA genes. The overall GC content of the chloroplast genome is 37.22%, wherein the corresponding values in the LSC, SSC, and IR regions are 35.3%, 30.79%, and 42.35%, respectively. Phylogenetic analysis showed that *P. canariensis* is sister to *P. dactylifera* with strong bootstrap support.

*Phoenix canariensis* Chabaud, Arecaceae, is one of the date palm species that grows in countries around the Arbian Gulf. Genus *Phoenix* contains about 19 species, distributed in southern Africa and Asia (Adawy et al. 2002; Jain et al. 2011). *Phoenix canariensis* Chabaud is used in an ornamental landscapes around the world (Antonio et al. 2017). It is also present in other continents in areas where the winter is temperate (Rousseau et al. 1999). For example, it is widely planted in parks and streets of China (Lan et al. 2020). It hybridizes naturally with the *Phoenix dactylifera* (date palm), but *P. canariensis* is stronger and has bigger leaves (Antonio et al. 2017). The plastid genome is valuable in plant systematics research due to its highly conserved structures, uniparental inheritance, and haploid nature (Fu et al. 2016). Plastid genome have also been smartly engineered to confer useful agronomic traits and/or serve as bioreactors (Jin and Daniell 2015). As one of the world’s most popular ornamental palms, the complete plastid genome of *P. canariensis* was sequenced, which will provide genomic and genetic sources for further research.

In this study, the complete plastome sequence of *P. canariensis* was reported and characterized. The fresh leaf sample of *P. canariensis* was acquired from one individual in Jinggangshan University (N 27°06′45.46ʺ, E 115°01′55.84ʺ), Ji’an, Jiangxi Province of China. The voucher specimen was kept in Key Laboratory of Ecological Environment and Resource Utilization, Jinggangshan University (JGSU20190826). Total DNA of *P. canariensis* was extracted from the fresh, young leaves (about 1.5 g) with a modified CTAB method (Doyle and Doyle 1987). The DNA library was prepared with a TruSeq DNA Sample Prep Kit (Illumina, USA) according to the instructions of the manufacturer. A genomic DNA library with an insert size of 400 bp was constructed and then sequenced by an Illumina HiSeq 2500 system. Approximately 5 Gb of sequences data were extracted from the total sequencing output and input into Organelle PBA (Soorni et al. 2017) to assemble the plastid genome. Annotation of the plastid genome was performed using the Dual Organellar GenoMe Annotator (DOGMA) online tool (Wyman et al. 2004) and Geneious v11.1.5 (Kearse et al. 2012)with *Washingtonia robusta* (GenBank: NC_029974.1) as the reference, then manually verified and corrected by comparison with *Phoenix dactylifera* (CM018784.1). Finally, we obtained a complete plastid genome of *P. canariensis* and submitted to GenBank with accession number (MT937173).

The chloroplast genome of *P. canariensis* shows a typical quadripartite structure of 158,477 bp p in full length, consisting of a large single copy region (LSC) of 86,189 bp, a small single copy region (SSC) of 17,704 bp, and a pair of inverted repeats (IR) regions of 27,292 bp inserted between LSC and SSC. The total GC content of plastome is 37.22%, with the corresponding values of 35.3%, 30.79%, and 42.35% in the LSC, SSC, and IR regions, respectively. The chloroplast genome comprises 132 genes, including 86 protein-coding genes, 8 ribosomal RNA genes, and 38 transfer RNA genes. 18 genes occurred in the IR region have two copies, including 6 protein-coding genes (rpl2, rpl23, rps7, rps19, ndhB, and ycf2), 8 tRNA genes (trnH-GUG, trnM-CAU, trnL-CAA, trnV-GAC, trnI-GAU, trnA-UGC, trnR-ACG, trnN-GUU), and 4 rRNA genes (rrn16, rrn23, rrn4.5, rrn5). There are 111 unique genes, among which 16 genes have one intron, and two genes, ycf3 and clp P, have two introns.

To analyze the phylogenetic position of *P. canariensis*, the maximum likelihood phylogenetic tree was generated based on the plastid genome of *P. canariensis* and other 19 species of the Arecaceae. Alignment was conducted using MAFFT v7.307 (Katoh and Standley, 2013). The phylogenetic tree was built using RAxML (Stamatakis 2014) with bootstrap set to 1000. *Sabal domingensis* (KF928963.1) served as the outgroup. The maximum likelihood（ML）phylogenetic tree suggested that *P. canariensis* is closely to *P. dactylifera* (Figure 1). The completion and characterization of the complete plastid genome sequence in this study provided helpful molecular resource for future phylogenetic and evolutionary studies of the valuable tree *P. canariensis*.

**Figure 1.**
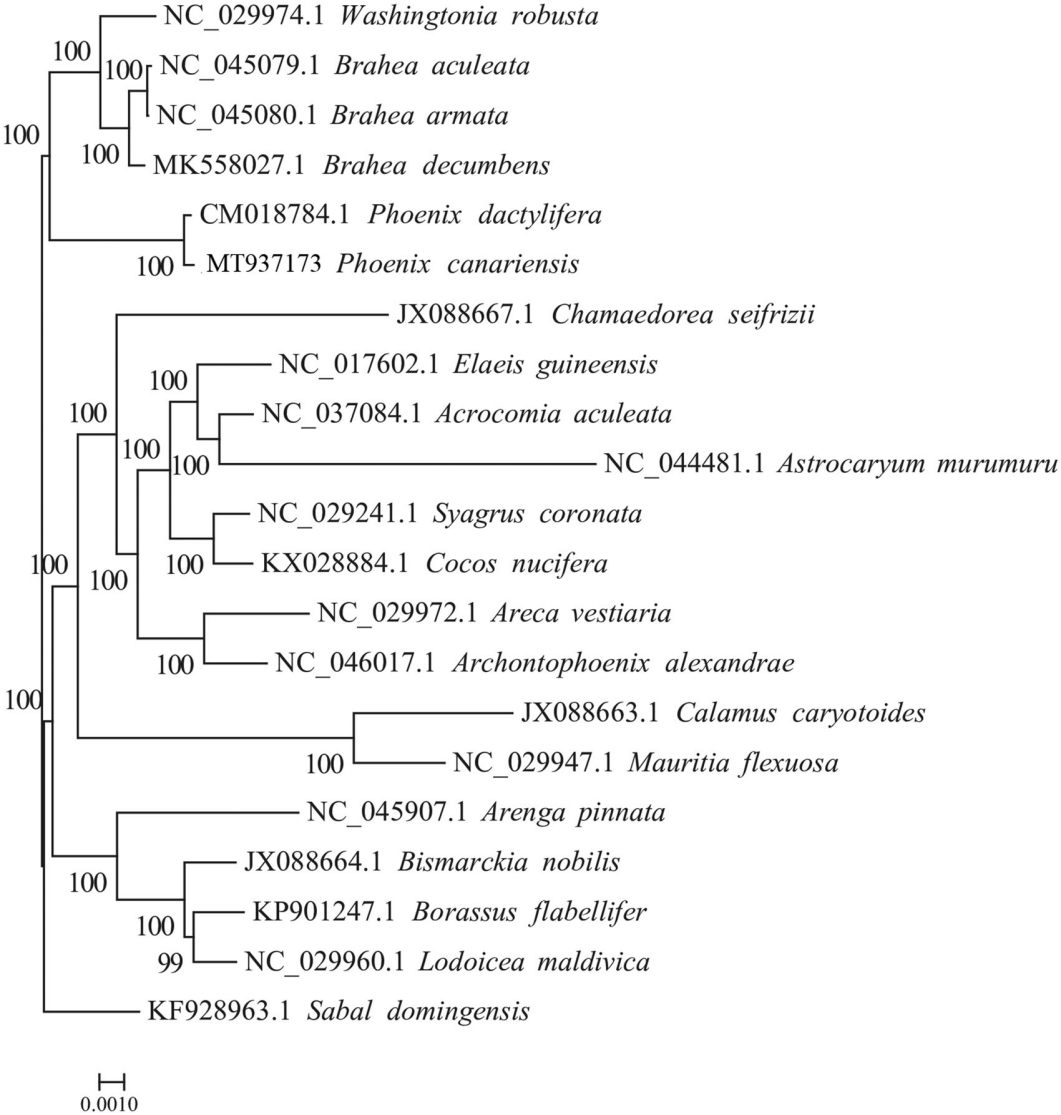
The maximum-likelihood (ML) tree based on the 20 representative plastid genomes of the Arecaceae. Numbers near the nodes mean bootstrap support value.

## Data Availability

The data that support the findings of this study are openly available in GenBank of NCBI at https://www.ncbi.nlm.nih.gov, reference number MT937173.
